# Osteosarcoma metastasis causing ileo-ileal intussusception

**DOI:** 10.1186/1477-7819-11-188

**Published:** 2013-08-12

**Authors:** Olivier Abbo, Kalitha Pinnagoda, Lionel A Micol, Maya Beck-Popovic, Jean-Marc Joseph

**Affiliations:** 1 Pediatric Surgery Unit, DMCP, Hôpital de l’Enfance, CHUV, Lausanne, Switzerland; 2 Pediatric Oncology Unit, DMCP, CHUV, Lausanne, Switzerland

**Keywords:** Osteosarcoma, Intussusception, Metastasis

## Abstract

Osteosarcoma metastasis causing intussusception is a very rare entity, with a pejorative prognosis. Based on a case, we performed a literature review in order to better assess this situation. We conclude that, in patients with a history of osteosarcoma lung metastasis, echographic and/or computed tomography scan evidence of a small bowel obstruction with intussusception should lead to an open surgical procedure if the laparoscopic approach does not allow to accurately explore and resect the lesion, in order to prevent misdiagnosis and to avoid further delay in the management.

## Background

Patient management of children with metastatic osteosarcoma has improved dramatically over the last few decades. This is thanks to an effective multidisciplinary approach associating multiple regimens of chemotherapy with surgical resection of primary and secondary lesions. Indeed, patients are living longer, and typical tumour evolution has changed. Hence physicians are having to deal with the involvement of different organs, even though such cases were considered exceptional in the 20th century [1,2]. Metastatic lesions of the bowel are not rare in adults, but this is generally as part of carcinomatosis [3]. Exceptionally, focal lesions of the intestinal wall can result in an acute intussusception. Based on a case, we performed a literature review to assess this rare problem in a child.

## Case presentation

A 17-year-old female patient was admitted having suffered 2 days of abdominal pain. She was in the 5th year of follow-up after an osteogenic osteosarcoma of the distal right femur. The primary tumor had been treated with three courses of induction chemotherapy using doxorubicine, methotrexate and cisplatin (following the AOST 0331 protocol) before she had undergone an amputation of the upper femoral shaft. Over the following years, she required two clam-shell thoracotomies for bilateral lung metastasis, along with chemotherapy (four courses of ifosfamid and VP16, and three courses of VP16 and cis-retinoic acid). The most recent intravenous treatment was administered 2 years ago; the last thoracic surgery was performed 36 months ago.

**Figure 1:**
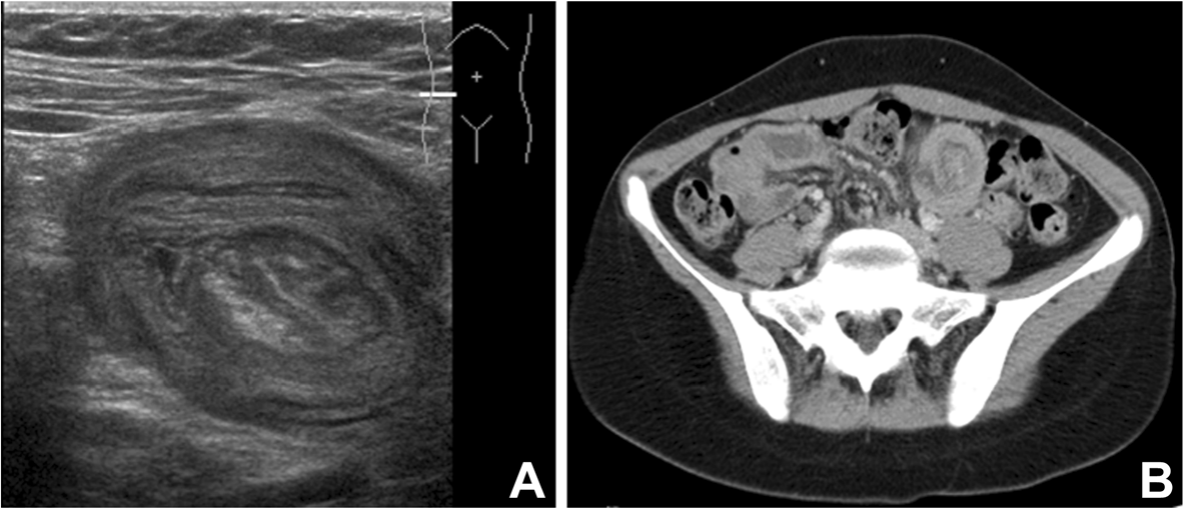
**Radiographic evidences of the suspected intussusception. (A)** Initial typical ultrasound demonstration of intussusception. **(B)** Computed tomography scan evidence of intussusception recurrence at day 5 after laparoscopic exploration.

The patient was admitted for mild abdominal pain; there was no vomiting or weight loss. Clinical examination revealed a slight tenderness of the right lower abdomen, without symptoms of intestinal obstruction. An initial ultrasound demonstrated the presence of an ileo-ileal intussusception (Figure 1A). After a short observation period, the lack of clinical improvement led to further investigations; a computed tomography (CT) scan confirmed the presence of an ileo-ileal intussusception. A magnetic resonance enterography was scheduled but, due the occurrence of occlusive symptoms, a laparoscopic exploration was performed. No intussusception was found despite a close examination of the entire small bowel with graspers.

The initial postoperative course was uneventful until the 4th day, when an acute bowel obstruction occurred. A nasogastric tube was placed. The persistence of symptoms led to another CT scan (Figure 1B) that revealed the recurrence of the ileo-ileal intussusception.

**Figure 2:**
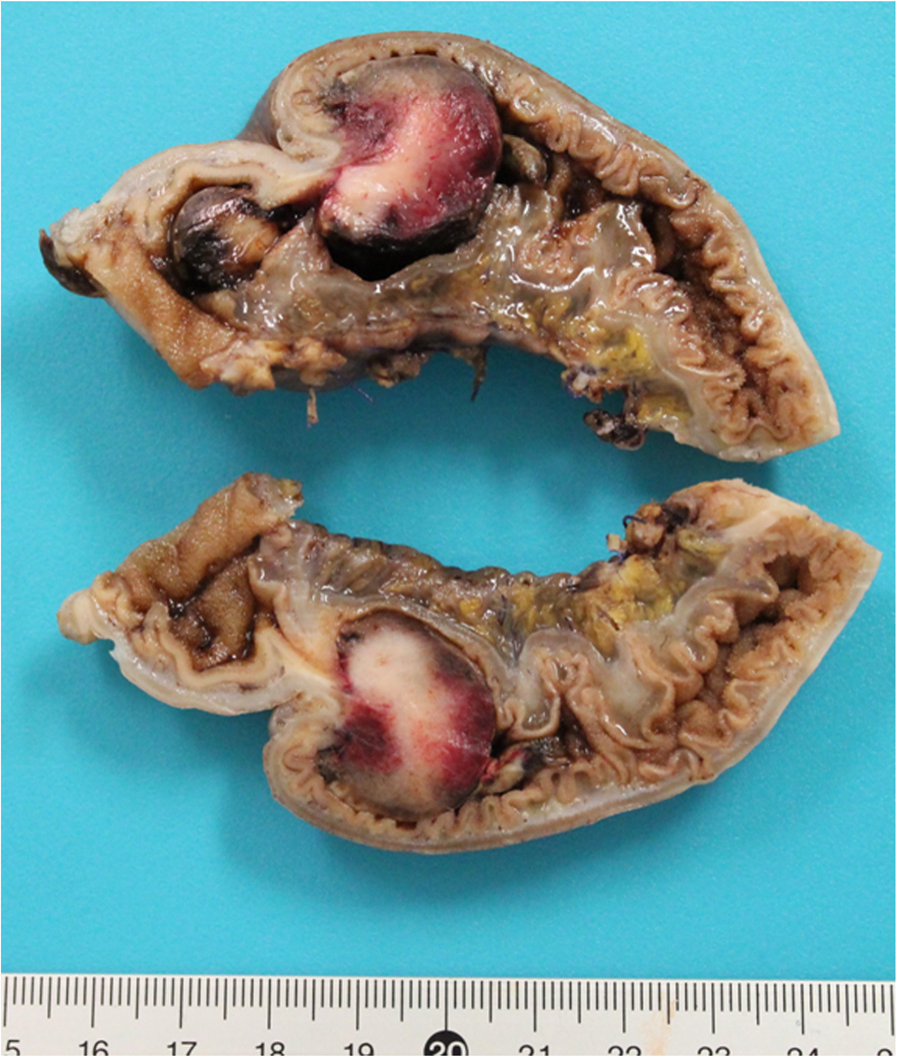
Macroscopic view of the specimen showing the intraluminal metastasis of osteosarcoma.

An open surgery was performed as the previous attempt at laparoscopy had failed to demonstrate the cause of the intussusception. The intussusception was easily found, but was irreducible due to a focal lesion of the intestine wall. A resection with end-to-end anastomosis was performed. The postoperative course was uneventful. Histopathology confirmed the metastasis from the initial tumor (Figure 2).

## Discussion

Intussusception is frequent and typically occurs in young children; it is mainly idiopathic or due to the reaction of lymphatic mesenteric nodes [4]. Less frequently, it is due to small bowel lesion - mainly malformations or benign tumors [5]. It is a rare entity in older children and adults, where other etiologies, such as embryonic rests and rare tumors, should be investigated [3]. Among tumors, most are primary*,* such as carcinomas or lymphomas, whereas others are secondary and rare. The characteristic origins of these metastases are lobular breast cancer, lung cancers, renal carcinomas and melanomas [4]. Osteosarcoma metastases have rarely been involved in such localization, with less than ten reported cases, mainly in adults [6]. Characteristically, metastases of osteosarcomas are found in lungs, bones, brain or liver. However, with the prolonged survival lengths and the enhanced efficacy of chemotherapies, even in children, unusual localizations are now reported, including the bowel. If carcinomatosis represents a classic entity in the evolution of solid tumors, focal lesions of the intestine wall are rare. The duodenum and the jejunum seem to be involved most of the time, while ileal, and recently gastric lesions, have only been reported in a few cases each [7]. Once the diagnosis of intussusception has been made, the classic reported and recommended approach is surgical resection, especially when there is a single metastasis [3]. In our reported case, the exact diagnosis was delayed due to an initial spontaneous resolution of the intussusception, probably during the anesthesia of the first surgical procedure. Despite a close examination of the bowel during this procedure, and the surgeon’s experience, the tumor was not detected. In similar cases, with the actual knowledge of possible metastatic lesion of osteosarcoma, one should consider in a second step extracting the bowel through a small incision during the same procedure in order to avoid missing these intra-luminal lesions. The principal benefit of the open procedure is to allow a meticulous palpation of the bowel, as is recommended for the lungs for osteosarcoma metastasis [2]. It has been well demonstrated in the latter that manual exploration of pulmonary parenchyma leads to a greater number of metastasis resections, which are known to be a major survival prognosis factor [8]. In these oncologic situations, and despite all the well known advantages of endoscopic surgery, the laparoscopic approach could be enhanced by a step approach.

Whatever the management of this patient, the prognosis is poor according to previously reported cases. This is probably due to the simultaneous or previous presence of lung metastasis in all cases, as reported by Horiuchi and colleagues [6]. A second explanation could be the time of diagnosis, where the patient has already undergone multiple regimens of chemotherapy without having avoided the spread of the disease in the circulatory system. Our patient’s palliative treatment is currently being considered with regard to the iatrogenic risk linked to further use of such therapeutics. Even if some general treatments are no longer available to such patients, surgery is required to solve acute obstruction and to try to control the metastatic disease in cases of a single secondary tumor [3].

## Conclusion

Osteosarcoma metastasis causing intussusception is a very rare entity, with a pejorative prognosis. In patients with a history of metastatic osteosarcoma of the lungs, the presence of a small bowel obstruction, together with echographic and/or CT scan evidence of intussusception, should lead to a manual exploration eventually associated with a preliminary laparoscopic approach, because of the need for a meticulous palpation of the lesion, potential resection, and the risk of misdiagnosing this entity.

## Consent

Written informed consent was obtained from the patient for publication of this Case report and any accompanying images. A copy of the written consent is available for review by the Editor-in-Chief of this journal.

## Abbreviations

CT: Computed tomography.

## Competing interests

The authors declare that they have no competing interests.

## Author’s contributions

All authors have been involved in the management of the patient and in the conception of the manuscript. OA, KP and JMJ have been involved in drafting the manuscript or revising it critically for important intellectual content. All authors read and approved the final manuscript.
